# Do movement-related beta oscillations change after stroke?

**DOI:** 10.1152/jn.00345.2014

**Published:** 2014-07-30

**Authors:** Holly E. Rossiter, Marie-Hélène Boudrias, Nick S. Ward

**Affiliations:** Sobell Department of Motor Neuroscience and Movement Disorders, UCL Institute of Neurology, London, United Kingdom

**Keywords:** stroke, motor cortex, beta oscillations, magnetoencephalography

## Abstract

Stroke is the most common cause of physical disability in the world today. While the key element of rehabilitative therapy is training, there is currently much interest in approaches that “prime” the primary motor cortex to be more excitable, thereby increasing the likelihood of experience-dependent plasticity. Cortical oscillations reflect the balance of excitation and inhibition, itself a key determinant of the potential for experience-dependent plasticity. In the motor system, beta-band oscillations are important and are thought to maintain the resting sensorimotor state. Here we examined motor cortex beta oscillations during rest and unimanual movement in a group of stroke patients and healthy control subjects, using magnetoencephalography. Movement-related beta desynchronization (MRBD) in contralateral primary motor cortex was found to be significantly reduced in patients compared with control subjects. Within the patient group, smaller MRBD was seen in those with more motor impairment. We speculate that impaired modulation of beta oscillations during affected hand grip is detrimental to motor control, highlighting this as a potential therapeutic target in neurorehabilitation.

stroke is the most common cause of physical disability in the world today. While the key element of rehabilitative therapy is training, there is currently much interest in approaches that “prime” the primary motor cortex (M1) to be more excitable, thereby increasing the likelihood of experience-dependent plasticity (Hagemann et al. 1998). Examples of this include noninvasive brain stimulation (NIBS) (Ayache et al. 2012; Hummel and Cohen 2006), active-passive bimanual movement therapy (APBT) (Stinear and Byblow 2004), and pharmacological interventions (Chollet et al. 2011; Molina-Luna et al. 2009). Understanding the mechanisms of these interventions and which stroke subtypes they work best in will help in delivering them to appropriate patients.

Currently, changes to M1 excitability can be assessed with transcranial magnetic stimulation (TMS), which measures the effect of experimental “priming” interventions on motor evoked potentials (MEPs) in the affected limb. One disadvantage of TMS is that it cannot be used in patients with absent MEPs in the paretic arm. Furthermore, the technique is most commonly performed at rest, which does not provide information about task-related changes in cortical excitability.

Previous studies exploring task-related activity after stroke have predominantly used fMRI. These studies have shown that patients with greater impairment tend to have more widespread activity across the motor network (including contralesional motor cortex) (Rehme et al. 2012; Ward 2011). One potential problem in using fMRI after stroke is that it relies on intact neurovascular coupling to generate the BOLD signal (Blicher et al. 2012). An alternative method of examining motor cortex activity that does not rely on neurovascular coupling is magnetoencephalography (MEG). MEG is an excellent technique for exploring the oscillatory dynamics in the cortex in more detail (Lopes da Silva 2013). Beta oscillations (15–30 Hz) are present during rest in M1 (Engel and Fries 2010). The strength of beta oscillations decreases just prior to a movement and then returns to a level above baseline after movement (Pfurtscheller and Lopes da Silva 1999). Movement-related beta power decrease (MRBD) has been linked to M1 excitability in recent EEG-TMS studies (Aono et al. 2013; Takemi et al. 2013), and both the strength and frequency of oscillations are influenced by levels of the inhibitory neurotransmitter GABA (Hall et al. 2010; Muthukumaraswamy et al. 2009, 2012). These results suggest that MEG might be a useful tool for studying the balance between inhibition and excitation in the human cortex.

Changes in beta-band oscillations have been seen in a number of settings. For example, enhanced beta oscillations have been seen as part of the aging process (Rossiter et al. 2014). Patients with Parkinson's disease exhibit abnormal beta oscillations in both basal ganglia (Kühn et al. 2004) and motor cortex (Heida et al. 2014) that they are unable to suppress when trying to initiate a movement. These findings have led to the suggestion that this abnormal beta-band activity is pathological and results in abnormal persistence of some sensorimotor states and therefore impairment of flexible motor control (Engel and Fries 2010). In stroke, previous studies have explored oscillatory parameters at rest and during tactile stimulation (Laaksonen et al. 2012; Tecchio et al. 2007a, 2007b), but currently there is little information on how these oscillations change during movement of affected limbs after stroke. It appears that beta oscillations may play a role in the pathology of diseases affecting movement and are therefore worthy of exploration in stroke.

In this study, we investigated cortical oscillatory signals at rest and during movement of the affected hand in stroke patients with a range of impairments and at different times after stroke. In stroke patients and older healthy control subjects, fMRI studies have shown task-related activation in both ipsilesional and contralesional M1 during unilateral hand movement (Rehme et al. 2012; Ward 2011). Bilateral MRBD with unilateral movement has been also seen in healthy control subjects with MEG (Jurkiewicz et al. 2006; Pfurtscheller and Lopes da Silva 1999). We therefore examined beta oscillations in motor cortices of both hemispheres. We hypothesized that beta oscillations would be diminished after stroke both at rest and during movement and that this would be more apparent in those with greater impairment.

## METHODS

### 

#### Subjects.

Twenty-five stroke patients (mean age 49 ± 13 yr, range 19–70 yr; 7 women, 18 men; 3 left-handed, 14 dominant-hand affected) participated (see Table 1 for more detailed demographic information). All patients suffered from first-ever stroke with weakness of at least wrist and finger extensors and hand interossei. We excluded patients with other neurological disorders, those unable to perform the grip task, those with metal implants likely to create artifacts in the MEG, and those with language/cognitive deficits sufficient to impair cooperation in the experiment. Thirty-two healthy participants (mean age 51 ± 21 yr, range 22–82 yr; 11 women, 21 men; 2 left-handed) took part in this study [results from this healthy cohort have been published separately (Rossiter et al. 2014)]. Full written consent was obtained from all subjects in accordance with the Declaration of Helsinki. The study was approved by the Joint Ethics Committee of the Institute of Neurology, UCL and National Hospital for Neurology and Neurosurgery, UCL Hospitals NHS Foundation Trust, London.

**Table 1.** Patient demographics and raw behavioral scores for affected hand

Patient	Sex	Age, yr	Affected Hand	Months After Stroke	Lesion Location	ARAT	NHPT, pegs/s	PCA	Grip, lb
*1*	Male	28	Dominant	4	Posterior MCA territory	28	0	−0.21	25
*2*	Male	51	Nondominant	6.8	Inferior MCA territory	57	0.47	0.15	58
*3*	Male	45	Dominant	72	Corona radiata/internal capsule	57	0.71	0.13	60
*4*	Male	53	Nondominant	4.1	Posterior MCA territory	20	0	0.23	8
*5*	Female	63	Nondominant	7.4	Corona radiata/internal capsule	57	0.58	−0.26	24
*6*	Male	56	Nondominant	1.6	Basal ganglia	57	0.23	0.18	24
*7*	Male	66	Nondominant	84.4	Inferior MCA territory	50	0.09	0.04	45
*8*	Male	39	Nondominant	16.3	Anterior MCA territory	0	0	−0.05	36
*9*	Male	70	Dominant	80.8	Corona radiata/internal capsule	30	0.01	−0.36	9
*10*	Male	54	Dominant	105.2	Corona radiata/internal capsule	56	0.29	−0.19	51
*11*	Female	30	Dominant	1.2	Corona radiata/internal capsule	57	0.74	0.06	44
*12*	Male	64	Dominant	76	Anterior MCA territory	54	0.04	0.24	54
*13*	Male	55	Nondominant	207.9	Anterior MCA territory	57	0.54	−0.05	17
*14*	Female	63	Dominant	0.9	Inferior MCA territory	57	0.59	0.16	37
*15*	Female	55	Nondominant	2.5	Thalamus	49	0.3	0.18	15
*16*	Male	54	Dominant	2.5	Inferior MCA territory	57	0.61	0.02	59
*17*	Female	19	Dominant	7.3	Basal ganglia	57	0.79	0.19	40
*18*	Male	51	Dominant	21.3	Anterior MCA territory	23	0	0.26	15
*19*	Male	52	Dominant	35	Inferior MCA territory	57	0.19	−0.24	64
*20*	Male	48	Nondominant	2.3	Posterior MCA territory	57	0.4	0.03	46
*21*	Male	46	Dominant	1.8	Anterior MCA territory	37	0	0.11	43
*22*	Male	59	Dominant	7.3	Anterior choroidal artery territory	57	0.7	−0.16	99
*23*	Male	37	Nondominant	2	Superior MCA territory	0	0	0.22	10
Mean		50 ± 13		32 ± 50		45 ± 19	0.32 ± 0.29		38 ± 22

MCA, middle cerebral artery; ARAT, Action Research Arm Test; NHPT, Nine-Hole Peg Test; PCA, principal component analysis. Mean ± SD values are also provided for age and behavioral scores.

#### Behavioral testing.

All patients were scored on the Nine-Hole Peg Test (NHPT) (Kellor et al. 1971; Oxford Grice et al. 2003), the Action Research Arm Test (ARAT) (Yozbatiran et al. 2008), and grip strength with a dynamometer. A principal component analysis (PCA) was performed on NHPT and ARAT in order to create a single motor impairment score unaffected by floor and ceiling effects in individual scores as has previously been done in our group (Ward et al. 2003a) (a lower PCA score corresponding to greater impairment).

#### Motor task.

Participants performed visually cued isometric hand grips with a manipulandum (Ward et al. 2006) during MEG recording. Prior to scanning, maximum voluntary contraction (MVC) was obtained for each subject. Patients used their affected hand for the task, whereas control participants used their dominant hand. Sixty trials were performed. The cue to perform a hand grip was the appearance of a “force thermometer” on the screen that provided continuous visual feedback about the force exerted. The target force was set at 30% of their MVC and displayed visually. Each grip was sustained for 3 s, with an interstimulus interval between 3 and 7 s. An identical manipulandum was placed in the inactive hand to check for mirror movements.

#### MEG recording.

MEG signals were measured continuously at 600 Hz during the task with a whole-head CTF Omega 275 MEG system (CTF, Vancouver, BC, Canada). Head localization was monitored continuously during the recordings in order to check for excessive movement. MEG data were preprocessed off-line with SPM8 (Wellcome Trust Centre for Neuroimaging, **www.fil.ion.ucl.ac.uk/spm**) (Litvak et al. 2011). Data were downsampled to 300 Hz and were filtered from 5 to 100 Hz. Data were epoched from −1 to +5 s, where *time 0* indicated onset of the visual cue. Trials with large eyeblinks or other artifacts were excluded.

#### Structural MRI recording.

A 3-T Siemens Trio scanner (Siemens, Erlangen, Germany) was used to acquire high-resolution T1-weighted anatomical images (1.3 × 1.3 × 1.3-mm voxels; 176 partitions, FoV = 256 × 240, TE = 2.48 ms, TR = 7.92 ms, FA = 16°). Structural MRIs could not be obtained in seven of the patients and four of the healthy control subjects because of MRI contraindications.

#### Data processing and analysis.

Lead fields were computed with a single-shell head model (Nolte 2003) based on an inner skull mesh derived by inverse-normalizing a canonical mesh to the subject's individual MRI image (Mattout et al. 2007). For subjects without individual MRIs, the canonical mesh was affine-transformed to fit their MEG fiducials. Coregistration between the MRI and MEG coordinate systems used three fiducial points: nasion and left and right preauricular. While acquiring the structural MRI, fiducial points were marked with vitamin E capsules in order to coregister with the MEG fiducials. The beamforming method is based on the linear projection of sensor data using a spatial filter computed from the lead field of the source of interest and the data covariance (Van Veen et al. 1997).

Oscillatory changes in the beta band (15–30 Hz) between rest and grip were localized with the Linearly Constrained Maximal Variance (LCMV) beamformer (Hillebrand and Barnes 2005; Vrba and Robinson 2001) as part of the SPM8 software. The passive time window was taken from −2.5 s to 0 s with 0 as the onset of the visual cue to move. The active time window was from 0.5 s to 3 s after the visual onset. The source signal was then extracted from the peak change in beta power that was found in M1 both contralateral and ipsilateral to the moving hand. The source orientation was in the direction yielding maximal signal variance. Morlet wavelet time-frequency analysis was used to explore the changes in beta across a trial from these locations. These were rescaled in order to show percent change from baseline (−1 to 0 s) and averaged across trials. The mean percent decrease in beta power (15–30 Hz) was then extracted from the 3-s movement period for each participant, and the percent beta decrease in contralateral M1 was divided by the percent beta decrease in ipsilateral M1 in order to create a ratio (MRBD ratio). The absolute baseline beta power (−1 s to 0 s) was also obtained. In total, nine different beta parameters were used for subsequent analysis: baseline beta (peak frequency and amplitude) from both motor cortices, MRBD (peak frequency and amplitude change) from both motor cortices, and MRBD ratio.

To determine differences in beta parameters between the two groups, we performed two-sample *t*-tests. To examine the relationship between beta parameters and impairment, we performed multiple linear regression, using beta parameters age, time since stroke, and absolute grip strength as explanatory independent variables. Grip strength and time since stroke were not found to explain any of the beta parameters in an exploratory stepwise regression and so were removed from subsequent analysis.

## RESULTS

All patients were able to perform the grip task adequately (the average force was 33 ± 3% for patients and 32 ± 1% for control subjects), and no mirror movements were observed in either group. Demographic information for the patients is detailed in Table 1.

**Fig. 1.**
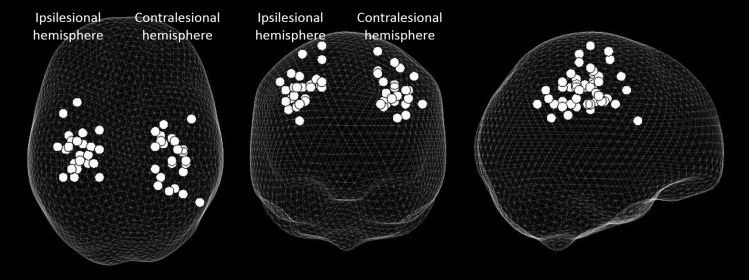
“Glass brain” showing peak change in beta power between rest and grip with affected hand only, in both ipsilesional and contralesional primary motor cortex (M1) (grip was performed with the right affected hand; left hand grips were flipped in the sagittal plane so that all data could be included on the same plot), with each dot representing an individual. The affected hemisphere is on the *left* and the unaffected hemisphere on the *right*. Results are displayed on a “glass brain” and shown from above (*left*), from behind (*middle*), and from the right side (*right*).

A change in beta power was seen between rest and grip in all participants in both contralateral and ipsilateral M1. The location of these peaks for the patient group can be seen in Fig. 1.

**Fig. 2.**
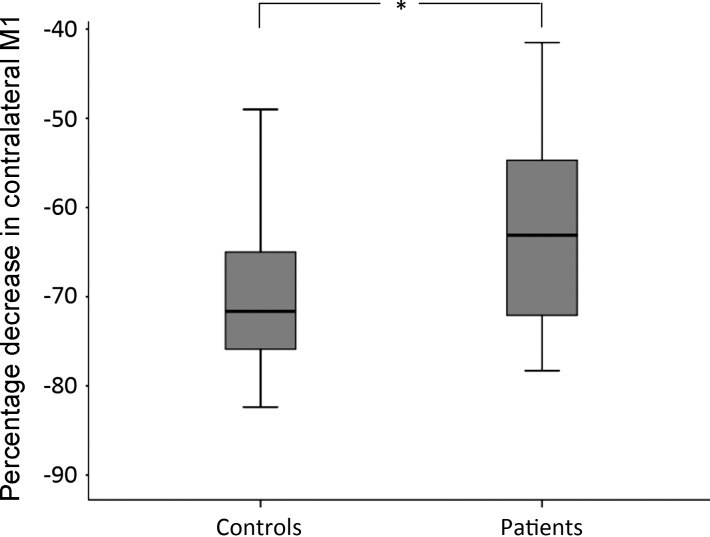
Box plot displaying % movement-related beta desynchronization (MRBD) in contralateral M1 in both the control group and the patient group. These were found to be significantly different with a 2-sample *t*-test (*P* = 0.005).

There was no significant between-group difference in baseline beta amplitude or frequency in either contralateral or ipsilateral M1. However, MRBD was significantly smaller in patients compared with control subjects in contralateral (2-sample *t*-test, *P* = 0.005) (Fig. 2) but not ipsilateral M1. The MRBD ratio was also significantly lower in patients compared with control subjects (2-sample *t*-test, *P* = 0.02). There was no between-group difference in frequency of peak MRBD in contralateral or ipsilateral M1.

**Fig. 3.**
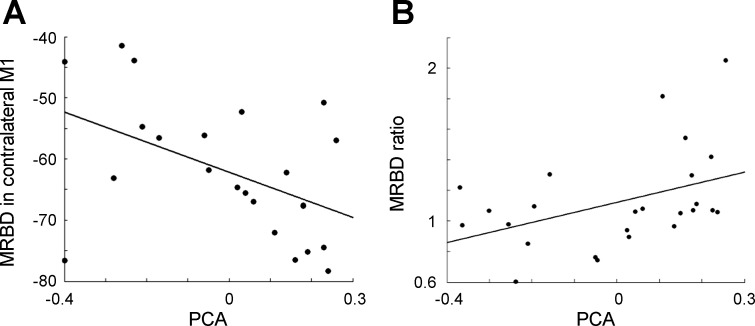
*A*: scatterplot showing % MRBD in contralateral M1 during grip compared with baseline against motor impairment score. There was a significant negative correlation between MRBD in contralateral M1 and motor impairment score (β = −0.52, *P* = 0.008). *B*: scatterplot showing the relationship between the MRBD ratio and motor impairment score. This correlation was significant (β = 0.42, *P* = 0.04). PCA is a motor impairment score derived from the principal components of the Nine-Hole Peg Test and the Action Research Arm Test. Higher PCA value equates to less impairment.

We then examined whether beta parameters correlated with motor impairment in the patient group. Neither power nor frequency of baseline beta oscillations, in either hemisphere, was related to motor impairment. However, MRBD in contralateral (but not ipsilateral) M1 correlated negatively with motor impairment [standardized regression coefficient (β) = −0.52, *P* = 0.008, *r*^2^ = 0.26]. In other words, there was a smaller reduction in beta power during affected hand grip in patients with more impairment. Furthermore, the MRBD ratio correlated positively with motor impairment (β = 0.42, *P* = 0.04, *R*^2^ = 0.17) (Fig. 3). Peak frequency of MRBD in contralateral and ipsilateral M1 did not correlate with impairment.

All significant correlations were found while controlling for age as a factor (age was included as a covariate in the multiple linear regression). None of the beta parameters was significantly explained by age alone.

## DISCUSSION

In this study, we investigated how motor cortex beta oscillations are affected in stroke patients with a range of impairments and at different times after stroke. We report two new key findings: First, contralateral (ipsilesional) M1 MRBD was diminished in patients compared with control subjects, although there was no difference in baseline beta power between groups. Second, in the stroke patients greater impairment was associated with lower contralateral (ipsilesional) M1 MRBD (and MRBD ratio). No difference was seen in baseline beta power between patients and control subjects. The only significant differences found were during dynamic changes across movement. This illustrates the importance of examining state-dependent dynamics during task-related movements, something that is better performed with MEG/EEG than with techniques such as fMRI and MRS, which have lower temporal resolution. Our results suggest that these oscillations may have an important role in the mechanisms of impairment of motor control after stroke.

fMRI studies have found more widespread activation in motor networks after stroke and specifically more bilateral recruitment of M1 (Ward et al. 2003b). With good recovery, this tends to revert back to a pattern similar to that seen in healthy control subjects, where the majority of the activation is seen in the contralateral M1 (Dijkhuizen et al. 2003; Ward et al. 2004). Our results indicate that those with more impairment have a more bilateral decrease in beta power across M1s during unilateral movement and those with less impairment have more “activity” (larger MRBD) in the contralateral M1, which is similar to the pattern seen in the above fMRI studies. Our results suggest a role for ipsilateral M1 MRBD during movement generation in those stroke patients with more impairment. Given that alterations in network connectivity have also been seen after stroke (Grefkes et al. 2008; Volz et al. 2014) it would be interesting to explore the relationship between oscillatory dynamics in different areas and levels of motor impairment.

While stroke patients and Parkinson's disease patients have very different pathologies, they both show a diminished MRBD in the motor cortex contralateral to the affected hand together with deficits in some aspects of motor control. Clearly the underlying pathophysiology in both conditions is different, but it may be that both groups lack the ability to modulate motor cortex beta power during movement, which then reduces their ability to generate volitional descending motor signals.

The amplitude of baseline beta power and MRBD have both been linked to levels of GABA (Gaetz et al. 2011; Hall et al. 2011; Muthukumaraswamy et al. 2012). The spectral characteristics of MEG data therefore have the potential to inform us about cortical inhibitory and/or excitatory processes that themselves are important determinants of the potential for experience-dependent plasticity (Benali et al. 2008). Furthermore, approaches to modeling these spectral data will allow inferences about synaptic physiology in humans in vivo (Bastos et al. 2012; Moran et al. 2009, 2011). Understanding the link between these oscillatory measures, the balance between inhibitory and excitatory processes, and the potential for experience-dependent plasticity is likely to be important in understanding the mechanisms of “priming” approaches such as NIBS and pharmacotherapy.

One of the limitations of this study is the variability in the patient group. The time since stroke in our cohort ranged from 1 mo to 17 yr (Table 1). To exclude the possibility that time since stroke affected the beta parameters, it was included in an exploratory stepwise multiple regression. It did not explain any of the beta parameters significantly, and we still saw a significant correlation with impairment in the patient group despite this variability. In future studies it would be of value to follow the same patient from the acute stage through to the chronic stage and see how these parameters alter with time and with recovery. This could tell us something about the timescale of alterations in poststroke plasticity and the window of opportunity for intensive rehabilitation (Carmichael 2012; Clarkson et al. 2010; Zeiler et al. 2013).

Furthermore, there was a range of ages in the patient group. We have previously reported that beta oscillations are affected by age in that the baseline beta power amplitude increases with advancing age (Rossiter et al. 2014). However, in this study age did not significantly account for any of the variability in the beta parameters when added to an exploratory stepwise multiple regression, suggesting that impairment explains the oscillatory dynamics over and above that due to any age effects.

There was also variability in the level of impairment in our patients, although this was something we were explicitly interested in. The amount of grip force an individual had to exert during scanning was scaled to his/her own MVC, which varied according to level of impairment. We chose to do this rather than use the same absolute force for everyone as it allowed us to study patients who were quite impaired and would otherwise not have been able to perform the task. To exclude the possibility that this variability in grip force may have contributed to our results (Mima et al. 1999), we included grip strength as a covariate in an exploratory stepwise multiple regression. Variation in the beta parameters was not explained by grip strength, and it was therefore removed from further analysis. The changes we see in the beta oscillations are unlikely to be accounted for by differences in absolute levels of performance.

One potential issue with this study is the variability in the peak beta coordinates and whether they can be definitively defined as M1 (Fig. 1). MRBD has been localized to M1 in a number of MEG studies previously (Gaetz et al. 2010; Hall et al. 2011; Jurkiewicz et al. 2006; Rossiter et al. 2014), and so it is expected that the largest change in beta would be within M1 during this motor task. Our group has also performed this exact same task in fMRI studies and found the largest peak of activation to be within contralateral M1 (Ward et al. 2003b, 2008; Ward and Frackowiak 2003); hence there is an expectation that these changes are in M1. There is also an important issue in MEG analysis relating to coregistration error, in terms of either head movement during scanning or errors in coregistration to MRI. As such, we cannot say with absolute certainty that our data relate to contralateral M1 in every case, but we feel this is the most likely scenario. Nevertheless, we felt it important to localize the peak beta change for each individual and use this coordinate for our further analysis.

In this study we chose to compare the affected hand of the patient group to the dominant hand of the control subjects. In a separate analysis, MRBD amplitude and frequency during grip from dominant hand were compared with nondominant hand in our group of healthy control subjects with *t*-tests, and no significant difference was found. Therefore we think it is appropriate to use the dominant hand of healthy control subjects for comparison with the affected hand of patients.

In summary, our results suggest that abnormalities in cortical oscillatory parameters may be an important part of the mechanism of motor impairment after stroke. Future work will be directed toward determining whether these oscillatory parameters reflect cortical inhibitory and/or excitatory processes and therefore represent biomarkers of the potential for experience-dependent plasticity in the poststroke brain.

## GRANTS

This research was supported by the European Commission under the 7th Framework Program-HEALTH-Collaborative Project Plasticise (Contract no. 223524) (**www.plasticise.eu**) (H. E. Rossiter), the Canadian Institutes of Health Research (M.-H. Boudrias), and The Wellcome Trust (N. S. Ward).

## DISCLOSURES

No conflicts of interest, financial or otherwise, are declared by the author(s).

## AUTHOR CONTRIBUTIONS

Author contributions: H.E.R., M.-H.B., and N.S.W. conception and design of research; H.E.R. and M.-H.B. performed experiments; H.E.R. analyzed data; H.E.R., M.-H.B., and N.S.W. interpreted results of experiments; H.E.R. prepared figures; H.E.R. drafted manuscript; H.E.R. and N.S.W. edited and revised manuscript; H.E.R., M.-H.B., and N.S.W. approved final version of manuscript.
